# Lyssavirus in Indian Flying Foxes, Sri Lanka

**DOI:** 10.3201/eid2208.151986

**Published:** 2016-08

**Authors:** Panduka S. Gunawardena, Denise A. Marston, Richard J. Ellis, Emma L. Wise, Anjana C. Karawita, Andrew C. Breed, Lorraine M. McElhinney, Nicholas Johnson, Ashley C. Banyard, Anthony R. Fooks

**Affiliations:** University of Peradeniya, Peradeniya, Sri Lanka (P.S. Gunawardena, A.C. Karawita);; Animal and Plant Health Agency, Addlestone, UK (D.A. Marston, R.J. Ellis, E.L. Wise, A.C. Breed, L.M. McElhinney, N. Johnson, A.C. Banyard, A.R. Fooks);; University of Liverpool, Liverpool, UK (L. M. McElhinney, A.R. Fooks)

**Keywords:** Gannoruwa bat lyssavirus, GBLV, rabies, lyssavirus, viruses, Indian flying fox, Pteropus medius, fruit bat, Asia, Sri Lanka

## Abstract

A novel lyssavirus was isolated from brains of Indian flying foxes (*Pteropus medius*) in Sri Lanka. Phylogenetic analysis of complete virus genome sequences, and geographic location and host species, provides strong evidence that this virus is a putative new lyssavirus species, designated as Gannoruwa bat lyssavirus.

There are 14 recognized species in the genus *Lyssavirus*: rabies virus (RABV), Lagos bat virus, Mokola virus (MOKV), Duvenhage virus, European bat lyssavirus types 1 and 2, Australian bat lyssavirus (ABLV), Aravan virus (ARAV), Khujand virus, Irkut virus, Shimoni bat virus, Bokeloh bat lyssavirus, West Caucasian bat virus, and Ikoma lyssavirus (IKOV) (*1*). RABV has a global distribution and is the dominant lyssavirus circulating in nonvolant (incapable of flight) mammals across Asia, including Sri Lanka. Bats are known reservoir hosts of all lyssaviruses except MOKV and IKOV. Discovery of new lyssaviruses in bats has stimulated research and surveillance efforts to identify additional members of this genus in bat populations (*2*).

Although lyssaviruses circulate in bats in Asia (*2*), RABV in bats in Asia remains unconfirmed. Irkut virus was the first bat lyssavirus identified in China (*3*). ARAV, Khujand virus, and West Caucasian bat virus have been isolated exclusively from insectivorous bats in Eurasia. Pathogen discovery in insectivorous and hematophagous bats is progressing. However, surveillance for lyssaviruses in fruit bats remains limited, particularly across Asia. Frugivorous bats in the Americas, which are distant genetically from bats of the family *Pteropodidae*, are independent reservoirs of RABV (*4*).

Although several regions contain fruit bats of the genus *Pteropus*, only pteropid bats in Australia have been identified as reservoirs for a lyssavirus species, ABLV, which has been isolated from all 4 *Pteropus* species in Australia. Moreover, ABLV has also been detected in at least 1 insectivorous bat (*Saccolaimus flaviventris*) (*5*). Although lyssavirus-specific antibodies have been detected in bats from several countries in Asia (*2*), the only lyssaviruses reportedly isolated from fruit bats in Asia have not been characterized (*6*,*7*).

In Sri Lanka, lyssavirus surveillance has focused on canine RABV as the primary public health concern. The Indian flying fox (*P. medius*, formerly known as *P. giganteus*), is a large frugivorous and nectarivorous bat that lives in forest, urban, and rural areas and is one of the most persecuted (e.g., cutting down of roosting trees and hunting) bats in southern Asia (*8*). These bats can fly long distances (<150 km) to forage and have a wide distribution (India, China, Bangladesh, Bhutan, Myanmar, the Maldives, Nepal, Pakistan, and Sri Lanka). We report identification of a lyssavirus in Indian flying foxes in Sri Lanka.

## The Study

Ethical clearance was obtained from the ethics committee of the Faculty of Veterinary Medicine and Animal Science at the University of Peradeniya (Peradeniya, Sri Lanka) and the Animal and Plant Health Agency (Addlestone, UK). Specimens were collected under permit no. WL/3/2/62/14 from the Sri Lanka Department of Wildlife Conservation.

During January 1, 2014–October 31, 2015, a total of 62 grounded bats were collected in an area inhabited by a long-established roost of ≈20,000 Indian flying foxes in Gannoruwa, Peradeniya, Sri Lanka (7°16′N, 80°36′E), which is located 600 m above sea level. Most bats were found dead. One bat (AK-42), which had clinical signs of illness, died shortly after capture (Table).

**Table:** Characteristics of 4 Indian flying foxes infected with Gannoruwa bat lyssavirus, Sri Lanka*

Original ID no.	APHA ID no.	Collection date	Location	Weight, g/age/sex	Clinical signs/condition	GenBank accession no.
AK-15	RV3266	2014 Sept 17	Peradeniya	1,500/mature/M	Dead at collection	KU244266
AK-40	RV3267	2015 May 8	Peradeniya	350/immature/F	Dead at collection	KU244267
AK-42	RV3268	2015 May 25	Peradeniya	500/immature/M	Cachectic, paresis, unable to fly, nystagmus, intermittent seizures (≈10 s), spontaneous vocalization, aggressiveness, biting, died shortly after capture	KU244268
AK-74	RV3269	2015 Sep 11	Gannoruwa	212.5/immature/F	Dead at collection	KU244269

*APHA, Animal and Plant Health Agency; ID, identification.

**Figure 1:**
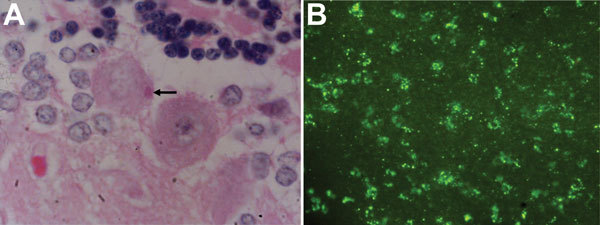
Negri bodies and lyssavirus antigens in brain tissue from an Indian flying fox, Sri Lanka. A) Degenerate Purkinje’s cell with an eosinophilic, intracytoplasmic inclusion body and a Negri body (arrow). Hematoxylin and eosin stain, original magnification ×1,000. B) Green fluorescence indicative of lyssavirus nucleoprotein in a brain smear subjected to a direct fluorescence antibody test with fluorescein isothiocyanate–conjugated monoclonal antibody. Original magnification ×100.

The first bat collected (AK-15) was a fresh carcass of a mature male that weighed 1.5 kg. A detailed necropsy showed that the animal had been healthy and had well-developed pectoral muscles. Except for a few multifocal hemorrhages in the lungs and mild, diffuse hyperemia and edema in the brain, gross pathologic findings were unremarkable. However, Negri bodies of various sizes were identified in the brain (Figure 1, panel A). Numerous aggregations of lyssavirus nucleocapsid antigen were observed in brain smears subjected to a direct fluorescence antibody test (dFAT) (Figure 1, panel B). Histopathologic examination of brain and spinal cord showed mild nonsuppurative lesions, leptomeningitis, and encephalomyelitis. Three additional dFAT-positive samples were identified from the 62 bats tested (Table). Subsequent virus isolation and molecular analysis were conducted for these 4 brain samples.

Virus was isolated by using N2A cells (*9*). After 5 days of incubation, 3 of 4 samples were positive for virus. Two of the isolates, RV3267 and RV3269, were subsequently cultured in BHK cells. RNA was extracted by using TRIzol reagent (Invitrogen, Paisley, UK). A pan-lyssavirus reverse transcription PCR yielded a specific 606-bp amplicon for the virus nucleoprotein gene (*10*). Results for a differential real-time reverse transcription PCR with a TaqMan probe specific for RABV showed no amplification for the 4 RNA samples. A specific 145-bp amplicon was visualized after electrophoresis on a 2% agarose gel. Thus, pan-lyssavirus primers used in the real-time assay detected this virus, but, the RABV-specific probe did not bind to the amplicon, which suggested presence of a non-RABV lyssavirus.

**Figure 2:**
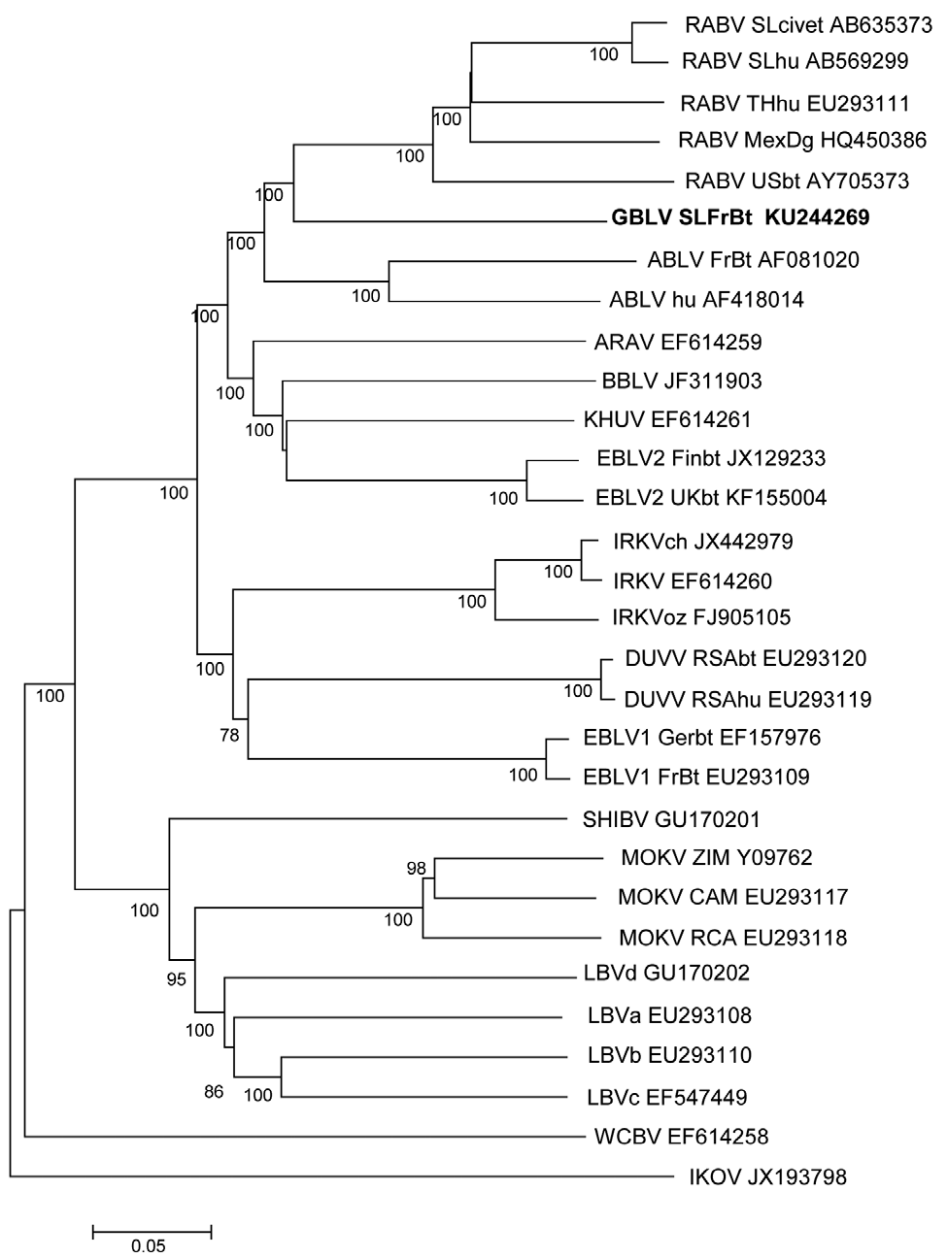
Phylogenetic relationships between representatives from all classified lyssaviruses and novel Gannoruwa bat lyssavirus (GBLV) on the basis of complete genome sequences. All 4 GBLV sequences form a monophyletic clade and are >99.9% identical across the genome; therefore, only 1 sequence (in bold) is shown. Relationships are shown as an unrooted phylogram, which was constructed by using the maximum-likelihood method and a general time reversible plus gamma distribution plus proportion of invariable sites model, and are identified by using the model test implemented in MEGA6 (http://www.megasoftware.net). Bootstrap values ≥70 (1,000 replicates) are indicated next to branches; sequences are listed with GenBank accession numbers. RABV, rabies virus; ABLV, Australian bat lyssavirus; ARAV, Aravan virus; BBLV, Bokeloh bat lyssavirus; KHUV, Khujand virus; EBLV, European bat lyssavirus; IRKV, Irkut virus; DUVV, Duvenhage virus; SHIBV, Shimoni bat virus; MOKV, Mokola virus; LBV, Lagos bat virus; WCBV, West Caucasian bat virus; IKOV, Ikoma lyssavirus. Scale bar indicates nucleotide substitutions per site.

Complete genome sequences (GenBank accession nos. KU244266–9) were obtained from brain RNA samples by using next-generation sequencing according to previous methods (*11*,*12*). Phylogenetic analysis of complete genome sequences, including representatives of all lyssavirus species, showed that sequences of the new non-RABV lyssavirus clustered with each other and had a common ancestor with ABLV and RABV in phylogroup 1 (Figure 2). This novel virus was designated as Gannoruwa bat lyssavirus (GBLV).

Representative canine and golden palm civet RABV sequences from Sri Lanka were included in the dataset, but those sequences clustered with other RABVs, distinct from the GBLV sequence (nucleotide identity 78%). Nucleotide identity across the complete genome ranged from 61% (IKOV) to 76.5% (ABLV), which showed that GBLV is a member of the genus *Lyssavirus* but is distinct from viruses circulating in nonvolant mammals in Sri Lanka.

## Conclusions

We report isolation of a novel non-RABV lyssavirus (GBLV) that is most closely related to RABV and ABLV. GBLV is pathogenic; it caused fatal disease in 4 Indian flying foxes, and clinical signs for these flying foxes were similar to those observed in other bat lyssavirus infections (Table). Diagnostic tests identified Negri bodies by staining with hematoxylin and eosin and lyssavirus antigens by dFAT in brain and spinal cord tissue (Figure 1). Molecular techniques identified lyssavirus nucleic acid, and full-genome analysis indicated that GBLV was divergent from known RABVs circulating in Sri Lanka (Figure 2).

Although rabies is prevalent in Sri Lanka, and a number of wildlife species have been confirmed as being rabid, most of the RABVs involved have not been genetically typed. Furthermore, over a 12-year period, only 1 bat tested for RABV was shown to be uninfected (*13*).

We report a novel non-RABV lyssavirus identified in Sri Lanka, which indicates that Indian flying foxes are a reservoir for lyssaviruses on the Indian subcontinent and nearby regions. Indian flying foxes are widespread in urban and rural areas and occasionally come in contact with humans and domestic dogs, which provides opportunities for virus spillover. Indian flying foxes are also reservoirs for Nipah virus in Bangladesh and India, where transmission to humans has resulted in outbreaks and human deaths. Other bat species, including insectivorous bats, might also be reservoir hosts for lyssaviruses in the study region. Thus, further surveillance is required to understand the role that bats play in the epidemiology of lyssaviruses in Asia.

Continued and extended surveillance of bats and other mammalian species is necessary to determine the distribution and prevalence of GBLV. Detailed phylogenetic analysis and monoclonal typing and antigenic mapping will help clarify the evolutionary relationship between GBLV and other lyssaviruses, in particular RABV and ABLV. In vitro and in vivo cross-neutralization and protection studies will elucidate properties of GBLV and provide information on protection from this virus by available prophylaxis.
